# Polycyclic aromatic hydrocarbon growth in a benzene discharge explored by IR-UV action spectroscopy†

**DOI:** 10.1039/d2cp01631a

**Published:** 2022-06-13

**Authors:** Alexander K. Lemmens, Daniël B. Rap, Sandra Brünken, Wybren Jan Buma, Anouk M. Rijs

**Affiliations:** Van't Hoff Institute for Molecular Sciences, University of Amsterdam Science Park 904 1098 XH Amsterdam The Netherlands; Institute for Molecules and Materials, FELIX Laboratory, Radboud University Toernooiveld 7 6525 ED Nijmegen The Netherlands sander.lemmens@ru.nl; Division of BioAnalytical Chemistry, AIMMS Amsterdam Institute of Molecular and Life Sciences, Vrije Universiteit Amsterdam De Boelelaan 1108 1081 HV Amsterdam The Netherlands

## Abstract

Infrared signatures of polycyclic aromatic hydrocarbons (PAHs) are detected towards many phases of stellar evolution. PAHs are major players in the carbon chemistry of the interstellar medium, forming the connection between small hydrocarbons and large fullerenes. However, as details on the formation of PAHs in these environments are still unclear, modeling their abundance and chemistry has remained far from trivial. By combining molecular beam mass-selective IR spectroscopy and calculated IR spectra, we analyze the discharge of benzene and identify resulting products including larger PAHs, radicals and intermediates that serve as promising candidates for radio astronomical searches. The identification of various reaction products indicates that different gas-phase reaction mechanisms leading to PAH growth must occur under the same conditions to account for all observed PAH-related species, thereby revealing the complex and interconnected network of PAH formation pathways. The results of this study highlight key (exothermic) reactions that need to be included in astrochemical models describing the carbon chemistry in our universe.

## Abstract 2

IR-UV spectroscopy of a benzene discharge reveals larger PAHs and intermediates showing that different gas-phase PAH growth mechanisms can occur under the same conditions. The identifications are promising candidates for radio astronomy searches.

## Graphical abstract

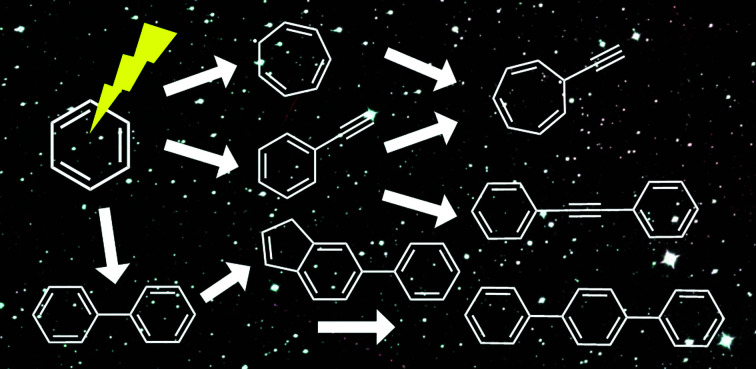

## A Introduction

The interstellar medium (ISM) can be considered as the repository of stellar and planetary matter. It contains the remains of previous stars and molecules that are continuously processed under widely varying conditions, such as temperature, density and external radiation (photons and cosmic rays) from stars and supernovae. More dense regions of the ISM can become gravitationally unstable and collapse to form new stars and planets.^1^ Within all these phases of the ISM (including planet-forming disks, surfaces of dark clouds and the diffuse ISM), infrared and microwave signatures of polycyclic aromatic hydrocarbons (PAHs) have been detected.^2–11^ The stability of the aromatic forms of hydrocarbons makes PAHs important carriers of organic matter. The evolution of PAHs in the ISM and their contribution to interstellar chemistry as constituents or catalysts is an important aspect of astrochemistry.^12^ They are expected to form a connection between small (aromatic) molecules such as benzene and benzonitrile and larger molecules such as fullerenes, both classes having been detected in cold regions of the ISM.^13,14^

Pyrolysis and crossed molecular beam studies have made major contributions towards understanding gas-phase formation pathways of PAHs.^15–17^ However, the complexity of the interconnected chemical pathways resulting in PAH formation and dissociation is still difficult to account for in astrochemical models.^18–20^ This generally leads to an underestimation of aromatic species in the ISM^21,22^ or in pyrolysis experiments.^23^ Identifying and mapping the bottom-up and top-down pathways of interstellar PAH formation and processing under the influence of UV photons,^24^ cosmic rays,^25^ or secondary electrons^26^ is key to improve our understanding of interstellar chemistry.^27^

**Fig. 1:**
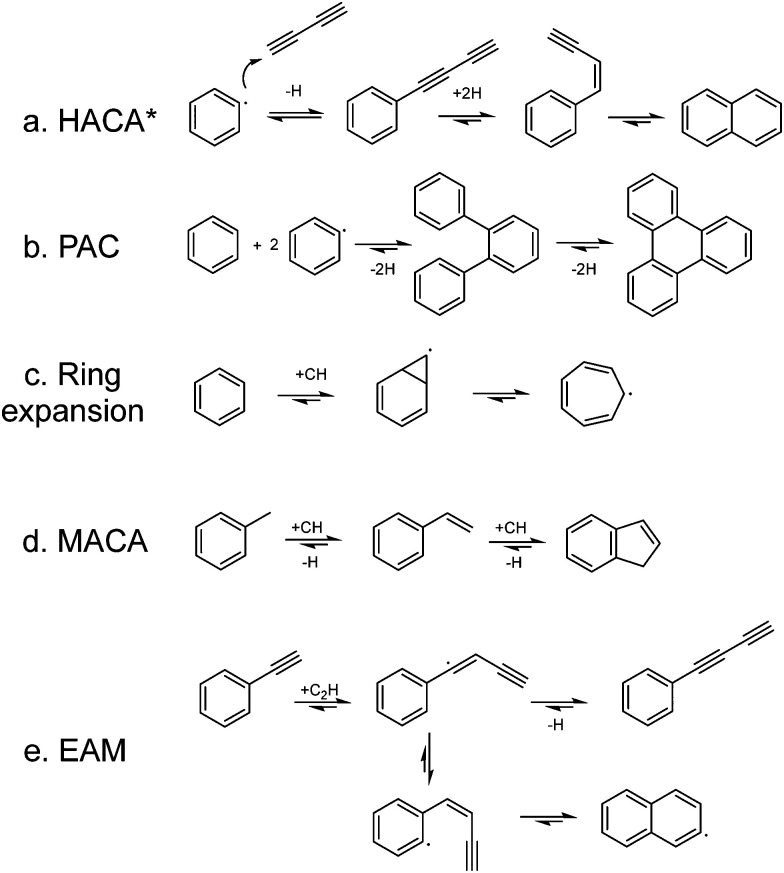
Reaction mechanisms leading to the formation of larger PAHs. The HACA and PAC mechanisms are initiated with the formation of a phenyl radical. The ring expansion, MACA and EAM mechanisms involve a methylidyne or an ethynyl radical. * The conventional HACA mechanism involves only acetylene, but here we classify mono-, di- and tri-acetylene addition in this mechanism.

Individual PAH growth reaction pathways that have been identified in crossed molecular beam or pyrolysis experiments (Fig. 1), generally start with the formation of a radical by hydrogen abstraction. In contrast to hotter environments such as circumstellar envelopes (CSE), UV radiation is expected to be responsible for the hydrogen abstraction in the cold ISM.^28–30^ A phenyl radical (possibly as part of a larger PAH) then reacts with a neutral hydrocarbon, which can be a small unsaturated hydrocarbon such as (mono-/di-/tri-/vinyl-)acetylene. This addition is followed by a cyclization step and together they are generally classified as the hydrogen-abstraction acetylene-addition (HACA, Fig. 1a) mechanism.^31–41^ The phenyl radical can also form a covalent bond with another aromatic ring resulting in biphenyl-type molecules, which is described by the phenyl addition cyclization (PAC, Fig. 1b) mechanism.^37,39,42,43^ Other mechanisms that have been identified and suggested as possible routes in the complex network of PAH formation pathways involve small radical hydrocarbons. These include the ethynyl (˙C_2_H) addition mechanism (EAM, Fig. 1e),^44^ the ring expansion mechanism with methylidyne (˙CH),^45–51^ (Fig. 1c), and the methylidyne addition–cyclization–aromatization (MACA, Fig. 1d) mechanism.^17^ Fast and successive radical–radical reactions have recently been added to the collection of possible reaction mechanisms.^52–54^

In the ISM, energetic particles or photons initiate gas phase chemistry by hydrogen abstraction.^24–26,55^ In our experiment, radicalization and fragmentation is realized by free electrons in an electrical discharge.^56^ Using this approach, a number of new species of astrochemical interest have been formed and identified by mass spectrometry^57–60^ or spectroscopy,^61–63^*i.a.* polyyne carbon chains or smaller cyclic hydrocarbons such as cyclopropenyl. Lee *et al.* have identified a wealth of chemical species resulting from the discharge of benzene using microwave spectroscopy.^64^ Although experimental conditions in such experiments differ from those found in cold regions of the ISM, identifying intermediates and products of discharge experiments is very useful as it can bring possible formation reactions to light that otherwise would remain elusive. With the support of quantum chemical calculations, exothermic and barrier-free reaction pathways can be revealed and the relevance of particular pathways under the cold and gas-phase conditions in the ISM can be evaluated.^28,56^

Microwave (MW) spectroscopy allows for determining relative abundances, thereby providing key information for benchmarking kinetic PAH growth studies.^18^ In the electrical discharge MW studies of benzene reported by Lee and coworkers,^64^ many products which are heavier in mass than the precursor have been identified. Since rotational spectroscopy relies on molecules possessing a permanent dipole moment, MW studies on hydrocarbon discharges in general reveal product species that contain chains or ring-chain combinations with a large dipole moment.^64,65^ Stable hydrocarbons, such as fully benzenoid PAHs, on the other hand, often only have a small or no dipole moment. To uncover the full complex network of PAH formation pathways, for which knowledge on such species is crucial, complementary analysis techniques are required. REMPI spectroscopy, as employed by Maier and co-workers, can be such method to identify products in the benzene discharge.^66,67^ We extend on this analysis and use a more general applicable identification method to significantly extend the product analysis of the benzene discharge.

In our studies, we use IR-UV action spectroscopy to record IR spectra in the fingerprint region of mass-selected products *in situ*.^68^ The UV laser used to ionize the molecules in the molecular beam is set to a wavelength where PAHs generally have large absorption cross sections, making our approach particularly sensitive to PAHs. Structural information is obtained by matching the measured IR absorption spectra to calculated spectra of candidate compounds.^69–72^ As will be discussed below, this approach enables us to identify many new species resulting from the complex network of PAH formation pathways starting with benzene that were, as of yet, not observable.

## B Methods

### Experimental

Experiments have been performed at the FELIX laboratory^73^ using a molecular beam spectrometer which is discussed in more detail in ref. 74. Argon (1.5 bar) was passed through a glass container containing benzene at room temperature. A gas pulse was created using a General Valve, which was discharged at 0.75 kV for about 100 μs between two ring electrodes with an opening of 3 mm and separated by 6 mm, traveling through a confined region for 6 mm before being expanded into a vacuum chamber. The design of the discharge nozzle was adapted from McCarthy *et al.*^62,63^ and has been described in more detail in ref. 56. A current of ∼20 mA was found to be optimal for the formation of the products of interest. The electron energy was estimated to be about 3 eV using the Paschen curve (with *γ* between 0.1 and 0.2^75^) and the relation between electron energy and pressure.^76^ The expanding gas pulse was skimmed before being crossed with three laser beams to perform IR-UV ion dip spectroscopy: one IR laser to probe the ground state vibrational levels and two UV lasers to perform 1 + 1′ REMPI. The IR light was provided by the free electron laser FELIX for spectroscopy between 400 and 1650 cm^−1^ with a bandwidth of 0.5–1% of the IR frequency. The UV excitation laser beam of 270 nm was provided by a Nd:YAG laser pumped dye laser (Spectra Physics, Lioptec) and the UV ionization laser beam of 193 nm by an ArF excimer laser (Neweks). The ions were detected in a reflectron time-of-flight mass spectrometer (Jordan Co.) equipped with a multichannel plate ion detector. The IR laser was run at half the repetition rate of the rest of the experiment in order to acquire alternating IR on and IR off shots, thereby compensating for shot-to-shot ion signal fluctuations. IR spectra are displayed as the logarithm of the ion signal depletion and are composed of an average of 90 shots per wavenumber, further averaged with a 5-point running average.

### Theoretical

Density functional theory calculations have been performed using the Gaussian 16 program.^77^ The B3LYP functional and N07D basis set,^69,78–80^ a combination that has been shown to perform well for predicting IR absorption spectra, has been used for structure optimization and harmonic vibrational frequency calculations. For comparison with the experimental spectra, theoretical spectra have been convoluted with a Gaussian function with a full width at half maximum of 1% of the photon frequency, corresponding to the FEL bandwidth. The minima and transition state structures have been optimized at the B3LYP/6-311G(d,p) level of theory. The transition states have been evaluated by intrinsic reaction coordinate (IRC) calculations to connect the minima on the potential energy surface.

## C Results

### Mass spectrometric overview

**Fig. 2:**
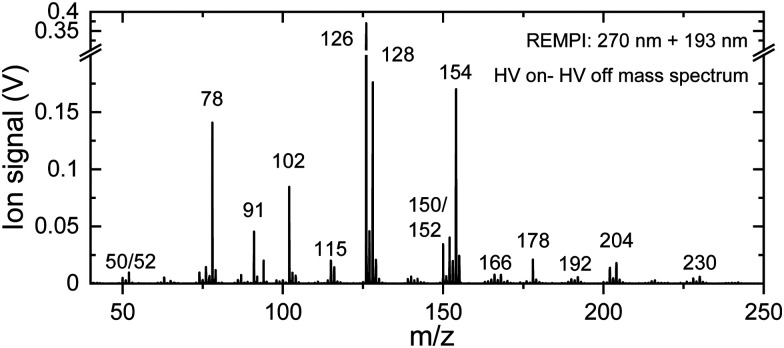
Mass spectra of fragments and products from the electrical discharge of benzene in argon. Neutral species are (singly) ionized using two-color (1 + 1′) REMPI with 270 and 193 nm. The spectrum displayed is obtained by subtracting the mass spectrum with the discharge off from the mass spectrum with the discharge source in operation (see Fig. S1 for the individual mass spectra, ESI†). To enhance the visibility of lower intensity signals the *y*-axis is truncated, the *m*/*z* 126 peak being twice as intense as the *m*/*z* 128 peak.

Fig. 2 shows the mass spectrum of the discharge products of benzene (*m*/*z* 78). Neutral products formed in the discharge are ionized using 1 + 1′ Resonance Enhanced MultiPhoton Ionization (REMPI), where the excitation laser is resonant with unresolved vibronic transitions of the formed molecules. The *m*/*z* 126 channel shows the strongest signal: twice the height of the *m*/*z* 128 peak (the *y*-axis is truncated for visibility of the lower intensity signals). Note that the signal strength does not need to reflect the abundance of species as UV absorption cross sections may vary for the different products and fragments in our experiment. We detect fragments of benzene down to *m*/*z* 50, including hydrogen abstracted species of benzene, as well as reaction products up to *m*/*z* 230. Because the UV excitation cross section at 270 nm is expected to be significantly lower for the smaller, no chromophore containing molecules, they are not observed in our mass spectrum. MW spectroscopy is more sensitive to such species and they have indeed been identified in a complementary study that was performed previously by Lee *et al.*^64^ Typically, 1 + 1′ resonant ionization does not result in doubly ionized species, *i.e.*, the *m*/*z* value corresponds to the mass of the products. The *m*/*z* 50 channel is assigned to C_4_H_2_, a common fragmentation product of PAHs that is likely to play a role in the formation of products with a higher mass than the parent species. Considering only its mass, the species with *m*/*z* 230 could correspond to a PAH consisting of multiple rings, showing that hydrocarbon growth occurs easily under discharge conditions. The exact chemical structures of the products can, however, not be deduced from merely their mass as multiple structural isomers exist for each mass. Here, we have used mass-selected IR action spectroscopy^56,69^ to obtain such structural information.

### IR spectroscopic assignments

**Fig. 3:**
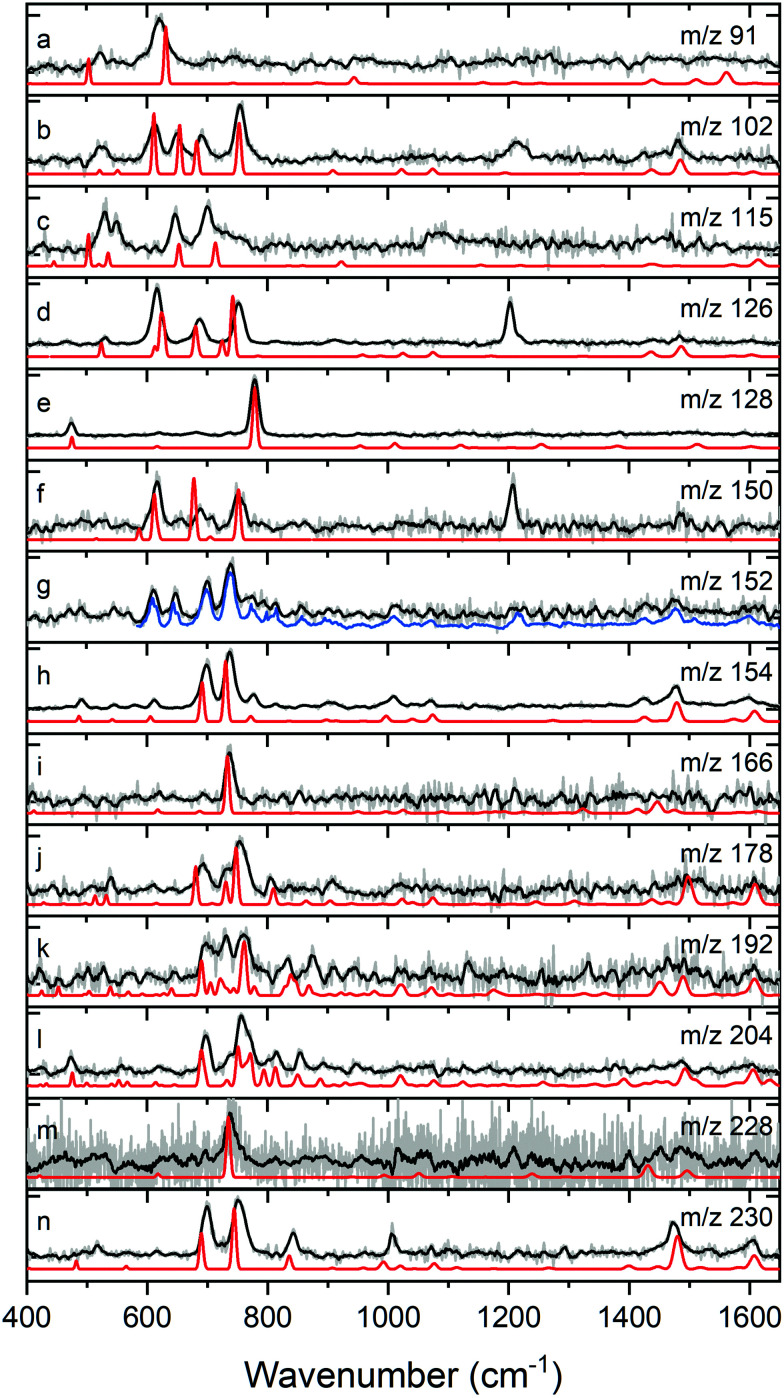
Assignment of molecular species formed in the discharge of benzene to the structures presented in Fig. 4a–m using mass-selective IR spectroscopy (black) with calculated IR absorption spectra (red). The mass channels shown are *m*/*z* = (a) 91, (b) 102, (c) 115, (d) 126, (e) 128, (f) 150, (g) 152, (h) 154, (i) 166, (j) 178, (k) 192, (l) 204, (m) 228 and (n) 230. The molecules assigned to each mass are (a) tropyl radical, (b) phenylacetylene, (c) tropylacetylene radical, (d) phenyldiacetylene, (e) naphthalene, (f) phenyltriacetylene, (g) 1- and 2-ethynylnaphthalene (1 : 2), (h) biphenyl, (i) fluorene, (j) diphenylacetylene and phenanthrene (2 : 1), (k) 1- and 2-phenyl-*H*-indene (2 : 1), (l) 1- and 2-phenylnaphthalene (1 : 1), (m) triphenylene and (n) *p*-terphenyl. For *m*/*z* 152, the blue trace consists for 50% of the FT-IR reference spectra of 1- and 2-ethynylnaphthalene (1 : 2) and for 50% of the experimental spectrum of *m*/*z* = 154.

Fig. 3 displays the experimentally obtained mass-selective IR spectra in the 400–1650 cm^−1^ range for all the significant products of the benzene discharge. For each of these masses we have calculated IR absorption spectra for structural isomers that reasonably can be expected and compared them with the experimentally obtained spectra. We find that the spectral region below 1000 cm^−1^ in combination with the mass of a species provides in general sufficiently unique spectral fingerprints to identify a molecular structure or a combination of structural isomers to be present in a particular *m*/*z* channel as discussed in the Methods section. As such, we come to the conclusion that *m*/*z* 91 must be assigned to the tropyl radical, *m*/*z* 102 to phenylacetylene, *m*/*z* 115 to the tropylacetylene radical, *m*/*z* 126 to phenyldiacetylene, *m*/*z* 128 to naphthalene, *m*/*z* 150 to phenyltriacetylene, *m*/*z* 152 to 1- and 2-ethynylnaphthalene (1 : 2), *m*/*z* 154 to biphenyl, *m*/*z* 166 to fluorene, *m*/*z* 178 to diphenylacetylene and phenanthrene (2 : 1), *m*/*z* 192 to 1- and 2-phenyl-*H*-indene (2 : 1) *m*/*z* 204 to 1- and 2-phenylnaphthalene, *m*/*z* 228 to triphenylene and *m*/*z* 230 to *p*-terphenyl.

In general, there is an excellent agreement between experimentally observed and theoretically predicted spectra, although the peak at 1210 cm^−1^ in *m*/*z* channels 102, 126, 150 and to a lesser extent 152 is not well predicted. This can be well understood as this feature is an overtone of the triple bond ≡CH deformation mode that appears around 616 cm^−1^ as was demonstrated in previous studies^56,81,82^ (see Fig. S2, ESI†). Since our calculations are based on the harmonic approximation, the overtone is not present in the predicted spectra. Although not predicted by the calculations, the peak at 1210 cm^−1^ serves as a useful diagnostic feature for ≡CH terminal groups. Similarly, the feature at 1090 cm^−1^ in the IR spectrum of the *m*/*z* 115 channel is expected to be an overtone of the same functional group (≡CH) with the fundamental ≡CH out-of-plane transition of the seven-membered ring being red-shifted to 550 cm^−1^ compared with a six-membered ring structure. More details of the assignments are discussed in Fig. S2–S13 (ESI†). The excluded options that are discussed in the ESI† illustrate how mismatches appear.

For several *m*/*z* channels (*m*/*z* 152, 178, 192 and 204) the experimental IR spectrum could not be the reproduced by a single isomer as not all IR signatures were accounted for by the predicted spectrum of a single species. In those cases, the spectra of more than one constitutional isomer were summed to achieve the best agreement between theory and experiment (see Fig. S8 and S10–S13, ESI†). The calculated spectra of diphenylacetylene and phenanthrene added in a ratio of 2 : 1 result in the best agreement with the experimental IR spectrum of *m*/*z* channel 178. For *m*/*z* 204, adding the calculated spectra of 1- and 2-ethynylnaphthalene in a 1 : 2 ratio leads to a good agreement with the experimental spectrum. The *m*/*z* 152 spectrum is somewhat more complicated as merely adding 1- and 2-ethynylnaphthalene in a 1 : 2 ratio does not lead to a satisfactory agreement. In this case, we recognize, however, the presence of an intense *m*/*z* 154 peak in the mass spectrum. This peak has been assigned to biphenyl which can easily lose molecular hydrogen upon photoionization resulting in an *m*/*z* of 152. IR absorption of biphenyl will lead to a reduction of *m*/*z* 154 ions and thus of *m*/*z* 152 ions. Overall, this will lead to the IR spectrum of the *m*/*z* 154 channel appearing as well in the *m*/*z* 152 channel. Indeed, we find that a predicted spectrum based for 50% on the FT-IR reference spectra of 1- and 2-ethynylnaphthalene (1 : 2) and 50% on the experimental spectrum of *m*/*z* = 154 reproduced the spectrum observed at *m*/*z* 152 much better.

## D Discussion

### Classification of identified products

**Fig. 4:**
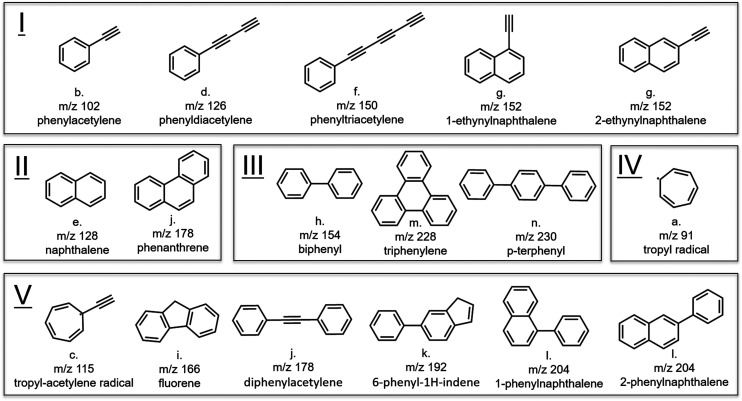
Molecular structures assigned to the mass-selected IR spectra in Fig. 3 with their respective *m*/*z* value. The letters a–n correspond to the IR spectra in Fig. 3.

The molecular structures of the discharge products assigned on the basis of their mass-selected IR spectra can be divided into 5 classes that are displayed in Fig. 4. We confirm the identification of the polyyne-substituted benzene molecules, so-called ring-chain molecules by Lee and coworkers:^64^ phenylacetylene, phenyldiacetylene and phenyltriacetylene (class I). These species are detectable by both MW and IR-UV spectroscopy as they are highly polar and at the same time have a large UV cross section at around 270 nm. The overlap in these identified species provides support for the reliability of both spectroscopic methods and confirms that the conditions in our discharge are comparable. Besides the polyyne-substituted phenyl species, two ethynylnaphthalene isomers (*m*/*z* 152) are newly identified ring-chain molecules in the product mixture of the benzene discharge. It is interesting to notice that two structurally related PAHs, *i.e.*, cyano-naphthalenes, have recently been detected *via* their rotational spectrum in TMC-1.^9^ In view of the similarities of the ethynyl and nitrile functional groups one might speculate that these compounds are formed along similar routes as the ethynylnaphthalenes. Observations in TMC-1 have shown that hydrocarbon cycles may contain both functional groups in different ratios.^83^ It would thus be highly interesting to perform similar experiments as done here with benzonitrile as a precursor. Class II comprises the unsubstituted PAH species naphthalene and phenanthrene, illustrating that fully benzenoid PAH growth readily occurs under our discharge conditions with only benzene as precursor. Class III is characterized by two intermediates and a product of the PAC mechanism: biphenyl and *p*-terphenyl or triphenylene, respectively.

Products with more than six carbon atoms in one ring, such as the seven-membered tropyl radical, form an interesting class of hydrocarbons (class IV). The identification of reactive radicals indicates that the discharge chemistry is stopped as the mixture is cooled and isolated in the adiabatic expansion of the molecular beam. Due to the isolated conditions of molecules in the molecular beam, the reaction mixture can be probed *in situ* without first collecting the reactants. The observation of these radical involving reactions corroborates the conclusions on the importance of radicals by Johansson *et al.*^84^ Class V contains hydrocarbons that are formed by a combination of the mechanisms summarized in Fig. 1. For example, the phenyl-naphthalene isomers are likely to be formed by the combination of the PAC and HACA reaction mechanisms, phenyl-indene *via* MACA and PAC reactions, tropylacetylene *via* ring expansion and HACA, and diphenylacetylene *via* HACA and PAC. The involvement of multiple pathways illustrates the complexity that needs to be considered in constructing astrochemical PAH formation models.

The formation of some of the products is surprising. For example, the diphenylacetylene signal is significantly more prominent than that of phenanthrene, yet diphenylacetylene is considerably higher in energy than phenanthrene (+194 kJ mol^−1^, zero-point vibrational energy corrected) as determined by DFT calculations (B3LYP/6-311G(d,p)^85^). Also other biphenyl species are generally higher in energy than their more compact counterparts, *e.g.* biphenyl with respect to acenaphthene (+17 kJ mol^−1^). The observation of species with a higher formation energy suggests that the reactions are kinetically controlled, similar to how pure gas-phase chemistry occurs in the ISM.

### Comparison to other gas-phase hydrocarbon chemistry experiments

The observation of the tropyl radical, the assignment of the PAHs naphthalene and phenanthrene (Class II), triphenylene, bi- and terphenyl (Class III), and the phenyl-substituted naphthalenes are excellent examples of the conclusion that microwave spectroscopy and mass-selective IR spectroscopy act as complementary techniques in the analysis of these complex mixtures. Combining these two techniques is thus an attractive means to fully reveal the composition of products and decrease the observational bias for particular species. Additionally, the two techniques each have their own merits in providing input for constructing chemical models. Microwave analysis, on the one hand, allows for the indirect determination of relative abundancies of highly polar ring-chain molecules, thereby providing the possibility to put quantified constraints on (astro)chemical models. Mass-selective IR-UV spectroscopy, on the other hand, is ideally suited for identifying (fully benzenoid) PAH molecules, which are of primary interest in PAH growth studies, because of their large UV absorption cross section.

The presence of 7-membered carbon rings was already suggested by Lee *et al.* because of the MW spectroscopic identification of tropone (C_7_H_6_O) in the discharge of benzene and oxygen.^64^ However, because it does not have a permanent electric dipole moment, the pure hydrocarbon analogue tropyl radical (C_7_H_7_) was not detected in their survey. The identification of the tropyl radical in this work, the evidence of formation of (methyl)tropyl,^50,67^ and the benzo[7]annulene radical in our previous work^56^ involving the discharge of naphthalene, highlights carbon insertion (Fig. 1c) as a possible hydrocarbon growth route. However, as was the case for the benzo[7]annulene formation in the naphthalene discharge, no intermediate for the tropyl radical was found in the benzene discharge. We expect that for larger PAHs, ring growth intermediates could very well be stabilized. Interestingly, no evidence was observed that would indicate the presence of the 6-membered ring isomer phenylpropargyl radical for *m*/*z* 115, which was previously identified in a benzene discharge (Fig. S5, ESI†).^86^ The role of methylidyne in the ring expansion mechanism confirms that besides the larger diacetylene and phenyl radicals also small radical hydrocarbons play an important role in PAH formation in our experiments.

In our previous study of the growth of naphthalene in a discharge, no biphenyl-type products were identified.^56^ Instead, the main products consisted of ring-chain hydrocarbons and larger PAHs. In the present study, however, a number of identified molecules contain covalently linked phenyl groups. This suggests that in the current experiments phenyl radicals – which react with benzene to form biphenyl structures – are formed abundantly. The presence of phenylacetylene and ethynyl-naphthalene implies at the same time that also (mono-/di-/tri-)acetylene and their radical forms are readily available, and not only phenyl radicals.

Similar to the discharge of naphthalene, a prominent species is phenyldiacetylene, which suggests a favorable route to PAH formation *via* diacetylene addition. Another similarity with the naphthalene discharge experiments is that no acenaphthylene, a common product in pyrolysis experiments,^87^ is detected. A large resemblance exists between our product mixture and the pyrolysis of azobenzene, as can be expected because in our mass spectrum we also detect phenyl radicals.^42^ However, the extensive fragmentation and subsequent recombination reactions in the discharge experiments result in an overall richer chemistry. The species that are formed include substituted PAHs with a large dipole moment, and these would be promising candidates for radio astronomical searches.

### The tropylacetylene radical case

**Fig. 5:**
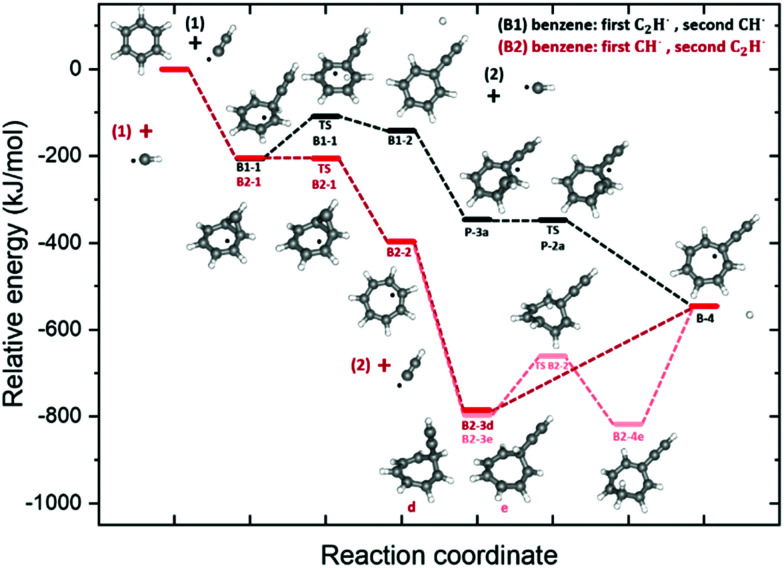
Potential energy surfaces of the benzene + ethynyl radical + methylidyne radical reaction pathways to tropylacetylene radical. The B1 pathway (black) involves first a reaction with the ethynyl radical to form B1-2 phenylacetylene and is followed by a reaction with a methylidyne radical. In the B2 pathway (red) the order is reversed and this pathway occurs *via* the B2-2 tropyl radical. The energies include the zero point vibrational energy correction and are calculated with respect to the entrance energy.

The large variety of products – including phenyl or acetylene substituted, 7-membered ring and ‘pure’ PAH species that are highlighted in the previous sections – indicates that various formation pathways are proceeding simultaneously in the benzene discharge. In order to investigate how and where multiple formation mechanisms are connected and lead to more complex hydrocarbons such as the ones in class V, we have constructed the potential energy surface of the formation of a typical product, the tropylacetylene radical. A combination of the ring expansion (see Fig. 1c) and acetylene addition mechanism (EAM, Fig. 1e) forms a logical pathway for this formation reaction. Both reaction sequences in which benzene reacts first with either the methylidyne radical (red pathways) or the ethynyl radical (black pathway) are shown in Fig. 5.

The (B1, black) pathway starts with ethynyl radical addition to form a benzene-ethynyl intermediate (B1-1). Hydrogen removal proceeds *via* (TS-B1-1) to yield the observed phenylacetylene (B1-2). In the next step, insertion of the methylidyne radical produces three similar bicyclic cyclopropa-phenylacetylene isomeric structures (P-3a,b,c) (see Fig. S14 in ESI†). The transition states (TS-P-2) have almost the same energy as the bicyclic minima (P-3, within ∼1–2 kJ mol^−1^). The formation of such bicyclic intermediate structures has been observed for the bimolecular reaction of methylidyne radical with styrene, where the corresponding transition states were also determined to be only 8 kJ mol^−1^ higher in energy.^17^

The (B2, red) pathway – starting with the addition of the methylidyne radical to form the tropyl radical (B2-2)-goes *via* a bicyclic cyclopropabenzene radical (B2-1) and a transition state (TS-B2-1). The latter is calculated to be only 2 kJ mol^−1^ higher in energy than B2-1, which is below the channel entrance energy.^49^ The subsequent addition of an ethynyl radical leads to an isomeric product distribution denoted by structures (d) and (e) as the seven-membered ring does not retain planarity. Both species proceed differently to the tropylacetylene product (B-4): the pathway *via* (e) shows hydrogen migration (TS-B2-2) on top of the aromatic surface prior to a barrierless removal of the hydrogen atom. The pathway *via* (d) to (B-4) occurs directly. As these different tropylacetylene adducts are both relatively low in energy and have a closed shell electron configuration, they may be stable and important reaction intermediates for ring growth and acetylene addition mechanisms. Two additional possible routes involving the phenyl radical are discussed in the ESI† (Fig. S14).

Both pathways of the combined EAM and ring expansion pathways are exothermic in nature as described in more detail in the ESI† (Fig. S14). Here, we have investigated possible routes towards a single isomer product, but in general the order of the reaction steps in the benzene discharge mixture will dictate the chemical complexity. Different sequences can form distinct conformers and isomers, such as the observed phenylacetylene intermediate (Fig. 3b) in the tropylacetylene example in Fig. 5. Each of the intermediate structures may act as a new precursor to extend the chemical complexity even more.

Many of the observed products possess a permanent dipole moment and are promising candidates for searches in the ISM by radio astronomy, especially the ring-chain molecules. For some species, rotational data are already available or could in principle be obtained. Their observation would shed light on the relevance of particular formation routes such as the ones presented in this study. Additionally, the determination of their relative abundance in astronomical objects would provide quantified observational constraints to chemical models.

## E Conclusions

Using mass-selective IR spectroscopy in the fingerprint region and quantum chemical calculations we have identified a large number of products in a benzene discharge, thereby revealing the complex and unexpectedly rich chemistry of benzene. By observing products in combination with (radical) intermediates, our experiments reveal multiple pathways to PAH growth to occur simultaneously and competitively. These pathways include the more commonly considered HACA and PAC mechanisms involving acetylene and phenyl radicals, respectively, but also the ring expansion mechanism that requires yet smaller radical hydrocarbons. Without laboratory input of possible structures and pathways such as provided in the present studies, important contributions to PAH chemistry can easily be overlooked. Our studies demonstrate that multiple routes to PAH formation explicitly need to be considered in order to account for the observed interstellar abundancies of PAHs. Quantum chemical calculations of the combined pathways to one of the reaction products provide a first insight into the energetic landscapes along different sequences of mechanisms that result in the same product. Many of these pathways show only submerged energy barriers, and are thus expected to proceed at low temperatures in the interstellar medium as well.

Our experiments have also identified intermediates and products that so far had not been observed or considered. A number of them – including the ring-chain hydrocarbons ethynyl-naphthalene and tropylacetylene – are of particular interest as they are quite polar and thus key targets for radio astronomy searches. Structurally similar to the recently detected cyano-naphthalenes, the astronomical observation and determination of the relative abundancies of these compounds would put constraints on specific structures for constructing quantitative PAH chemistry models. Laboratory experiments such as the one reported here – in which it is brought to light how the complex network of PAH formation pathways is interconnected – in combination with observational constraints are the way forward to disentangle the complex carbon chemistry in the universe.

## Author contributions

A. K. L., D. B. R. and A. M. R. conceived and designed the experiments. A. K. L. and D. B. R. performed the experiments and computations. The results were interpreted and described in this article by A. K. L., D. B. R., S. B., W. J. B. and A. M. R.

## Conflicts of interest

There are no conflicts to declare.

## Supplementary Material

CP-024-D2CP01631A-s001
