# Supplementary material for: Polycyclic aromatic hydrocarbon growth in a benzene discharge explored by IR-UV action spectroscopy
Source: Phys Chem Chem Phys. 2022 Jun 13;24(24):14816–24. doi: 10.1039/d2cp01631a (PMC9215700; doi:10.1039/d2cp01631a)
Supplement: CP-024-D2CP01631A-s001 [file CP-024-D2CP01631A-s001.pdf]

Electronic Supplementary Material (ESI) for Physical Chemistry Chemical Physics.
This journal is © the Owner Societies 2022

Supplementary information

Polycyclic aromatic hydrocarbon growth in a benzene discharge explored by IR-UV action spectroscopy

Alexander K. Lemmens*, Daniël B. Rap*, Sandra Brünken, Wybren Jan Buma, Anouk M. Rijs

*equal contribution to publication

Corresponding author: sander.lemmens@ru.nl

Mass spectra: Supplementary Figure 1 shows two mass spectra of benzene with discharge on and off. Comparison of the two spectra clearly shows the compounds that are formed during this discharge.

IR spectra: In our spectra we observe a characteristic vibrational band around 1200 cm^{-1} that is assigned to molecules that contain an ethynyl (-C≡CH) group. This feature is thought to be due to anharmonic effects and is assigned to the overtone of the -C≡CH out-of-plane mode which is typically located around 600 cm^{-1} [1–3]. Such an assignment is supported by comparing the experimental spectrum of m/z 126 and an anharmonic and harmonic calculation of the IR absorption spectrum of phenyldiacetylene as shown in Supplementary Figure 2. This Figure clearly shows that the 1200 cm^{-1} indeed arises from anharmonicity.

Besides the assignment of particular molecules, the comparison of experimental spectra and calculated infrared spectra of multiple molecules also has enabled to exclude certain species. A detailed analysis of the experimental spectra is shown in the Supplementary Figures 3-13.

Reaction pathway: An additional reaction pathway starting from the phenyl radical and accompanying analysis is shown in Supplementary Figure 14.

Mass spectra of the benzene discharge

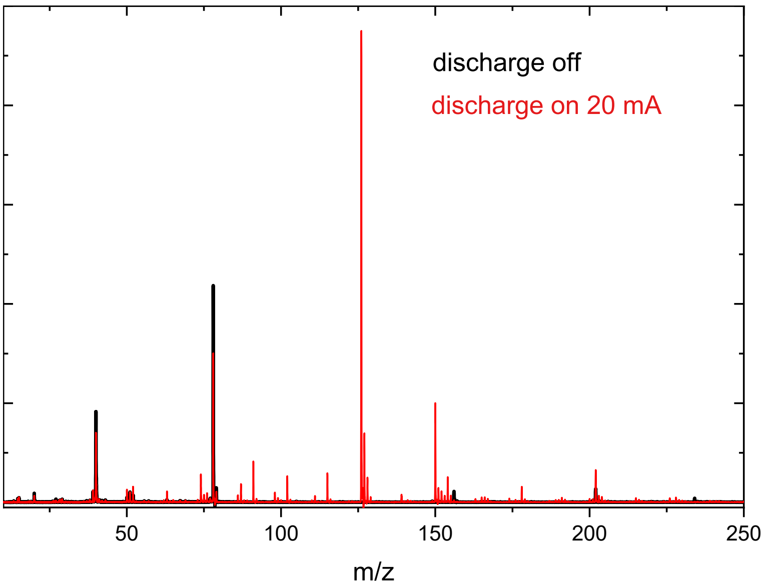

Supplementary Figure 1: Experimental mass spectra of discharge off (black) and discharge on (red). The masses of the parent (m/z 78) and argon (m/z 40, carrier gas) show a reduction upon discharge. Neutral species are (singly) ionized using two-color REMPI with 270 and 193 nm.

Anharmonic calculation accurately predicts the 1210 cm^{-1} feature

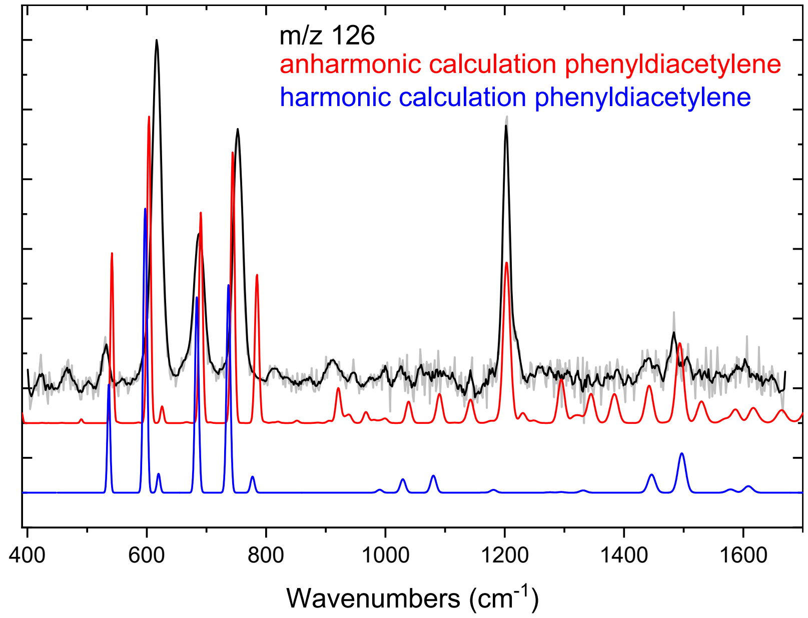

Supplementary Figure 2: Comparison of experimental spectrum of m/z 126 and the harmonic and anharmonic calculations of phenyldiacetylene. Black trace: experimental infrared spectrum of m/z 126 formed in a benzene discharge. Red trace: calculated anharmonic infrared spectrum of phenyldiacetylene. Blue trace: calculated harmonic infrared spectrum of phenyldiacetylene. The B3LYP functional in combination with the Jun-cc-pVDZ basis set has been used. Particular vibrations have been selectively removed from the anharmonic treatment to avoid large and incorrect shifts in some of these fundamental frequencies. The anharmonic calculation correctly predicts the overtone of the C-H out of plane bending mode observed in the experimental spectrum at 1210 cm^{-1}.

Detailed analysis of the experimental infrared spectra

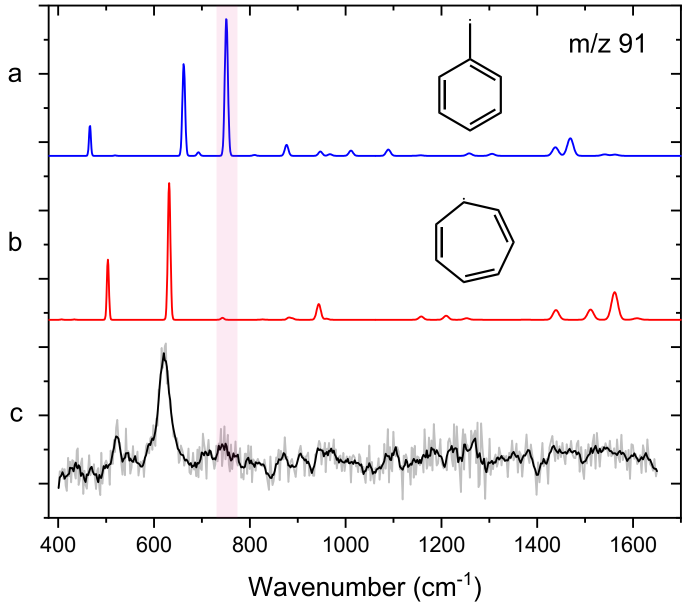

Supplementary Figure 3: Comparison of calculated infrared spectra of possible isomers for the m/z 91 channel to the experimental IR spectrum. The calculated spectrum of the tropyl radical (red, b) is assigned to the experimental spectrum of m/z 91 (black, c). The presence of benzyl radical (blue, a) is excluded based on the absence of intensity around 750 cm^{-1} as shown by the light-red shaded band.

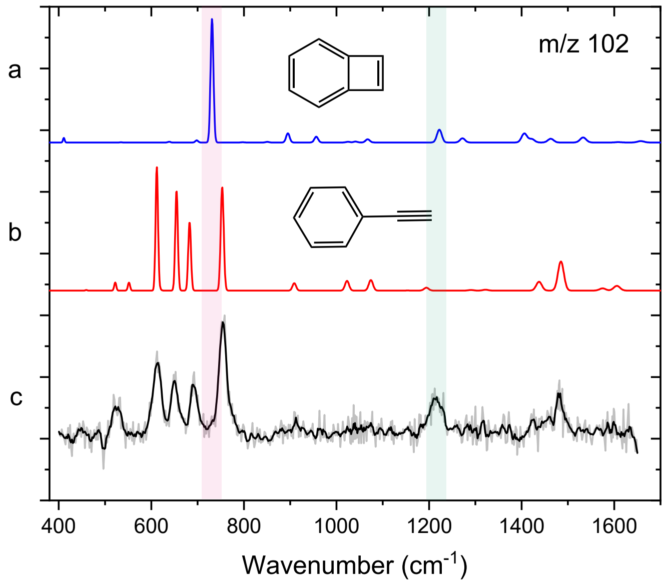

Supplementary Figure 4: Comparison of calculated infrared spectra of possible isomers for the m/z 102 channel to the experimental IR spectrum. The calculated spectrum of phenylacetylene (red, b) is assigned to the experimental spectrum of m/z 102 (black, c). The overtone at 1210 cm^{-1} observed in the experimental spectrum (light-green shaded band) is indicative for an ethynyl (-C≡CH) group and supports the assignment of phenylacetylene to m/z 102. The closed ring structure (blue, a) is excluded based on the fact that the most intense predicted band is not observed in the experimental spectrum (light-red shaded band).

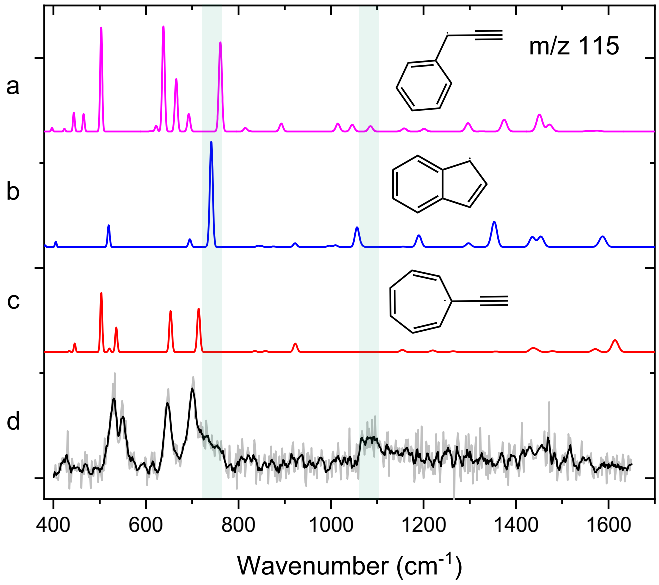

Supplementary Figure 5: Comparison of calculated infrared spectra of possible isomers for the m/z 115 channel to the experimental IR spectrum. The calculated spectrum of tropyl-acetylene radical (red, c) is assigned to the experimental spectrum of m/z 115 (black, d). The overtone around 1080 cm^{-1} observed in the experimental spectrum (light-green shaded band) is indicative for an ethynyl (-C≡CH) group and supports the assignment of tropyl-acetylene radical to m/z 115. A small contribution of the closed ring structure indenyl radical (blue, b) is observed in the shoulder of the experimental band around 750 cm^{-1} (light-green shaded band). The phenylpropargyl radical (pink, a) is excluded based the missing doublet at 550 cm^{-1} and the mismatch in band separation for the experimental bands at 646 and 700 cm^{-1} versus the calculated at 637 and 760 cm^{-1} (which are in better agreement with the tropyl-acetylene calculation).

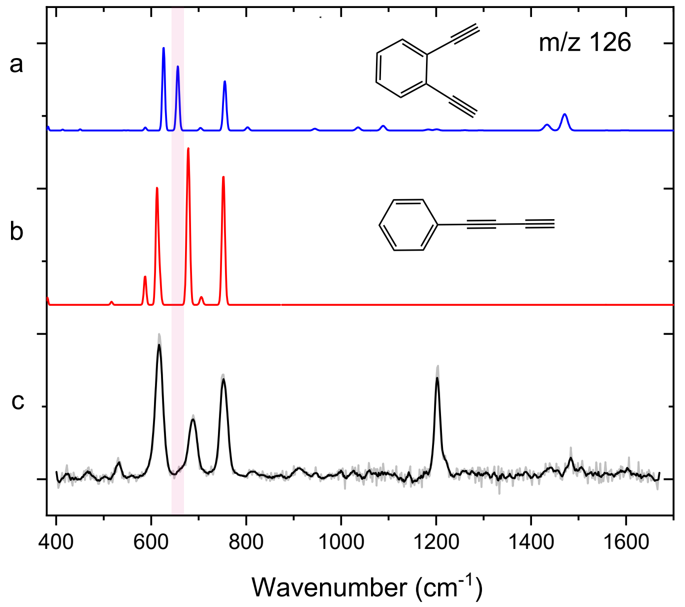

Supplementary Figure 6: Comparison of calculated infrared spectra of possible isomers for the m/z 126 channel to the experimental IR spectrum. The calculated spectrum of phenyldiacetylene (red, b) is assigned to the experimental spectrum of m/z 126 (black, c). The overtone at 1210 cm^{-1} observed in the experimental spectrum (light-green shaded band) is indicative for an ethynyl (-C≡CH) group and supports the assignment of phenyldiacetylene to m/z 126. From the assignment of m/z 102 (Supplementary Figure 4), we determine that a 30 cm^{-1} shift for modes other than the -C≡CH out of plane vibration is unlikely and therefore we can exclude additional contribution of di-ethynyl-benzene isomers by the incorrect peak spacings (light-red shaded band) in the lower wavenumber region, for which 1,2-diethynyl-benzene (blue, a) has been taken as an example molecule.

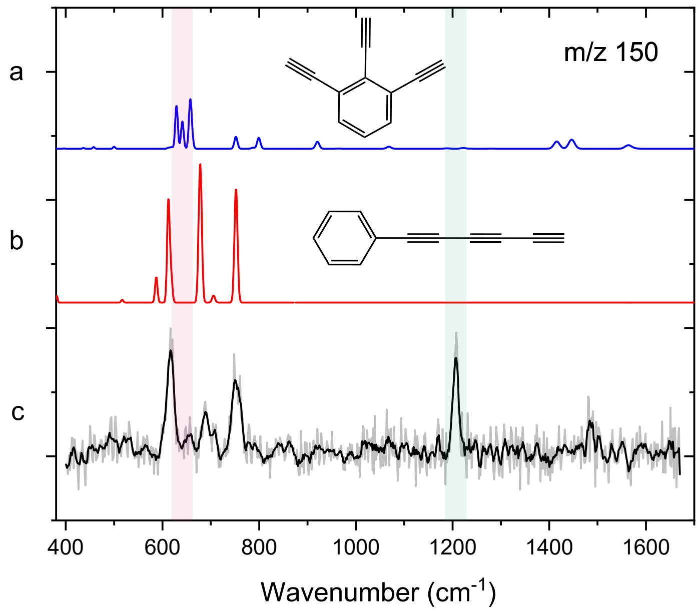

Supplementary Figure 7: Comparison of calculated infrared spectra of possible isomers for the m/z 150 channel to the experimental IR spectrum. The calculated spectrum of phenyltriacetylene (red, b) is assigned to the experimental spectrum of m/z 150 (black, c). The overtone at 1210 cm^{-1} observed in the experimental spectrum (light-green shaded band) is indicative for an ethynyl (-C≡CH) group and supports the assignment of phenyltriacetylene to m/z 150. 1,2,3-triacetylene-benzene is excluded based on the incorrect peak spacing in the lower wavenumber region (light-red shaded band). Both the calculated spectra of phenyldiacetylene (Supplementary Figure 6) and phenyltriacetylene show an intensity discrepancy for the band around 700 cm^{-1}, but this does not interfere with the assignment.

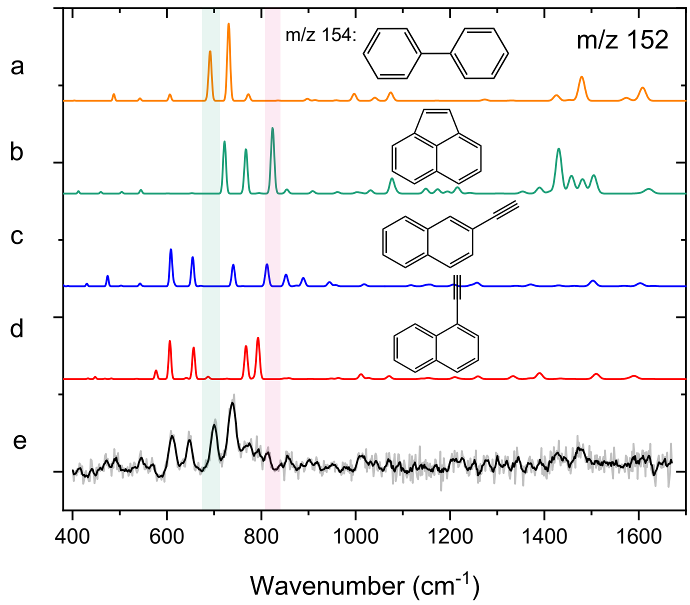

Supplementary Figure 8: Comparison of calculated infrared spectra of possible isomers for the m/z 152 channel to the experimental IR spectrum. The calculated spectra of 1-ethynylnaphthalene (red, d) and 2-ethynylnaphthalene (blue, c) are assigned in a 1:2 ratio to the experimental spectrum of m/z 152 (black, c) to account for 50 % of the signal. Additionally, the other 50 % is explained by the band of biphenyl (m/z 154, orange, a) shown in the light-green shaded band. This is the result of molecular hydrogen loss due to photo-fragmentation of biphenyl (assigned to the m/z 154 channel) upon photoionization. The presence of acenaphthylene (green, b) is excluded on the basis of the missing band highlighted by the light-red shaded band.

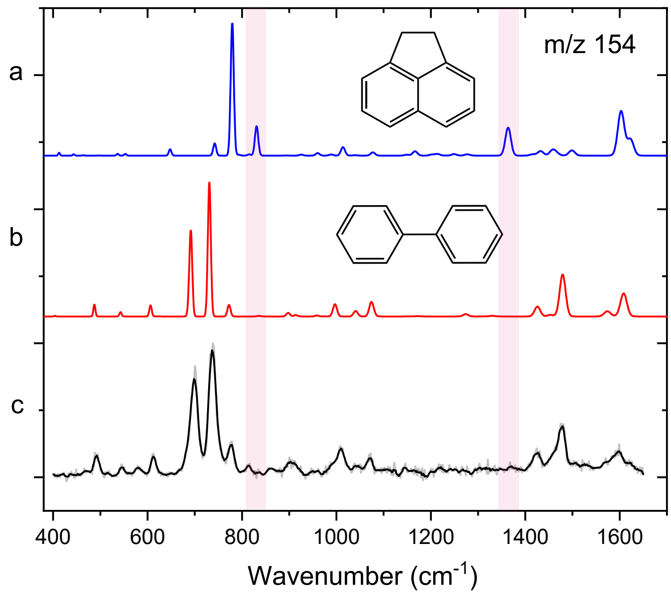

Supplementary Figure 9: Comparison of calculated infrared spectra of possible isomers for the m/z 154 channel to the experimental IR spectrum. The calculated spectrum of biphenyl (red, b) is assigned to the experimental spectrum of m/z 154 (black, c). The presence of the acenaphthene molecule (blue, a) is excluded based on the missing peaks in the light-red shaded bands.

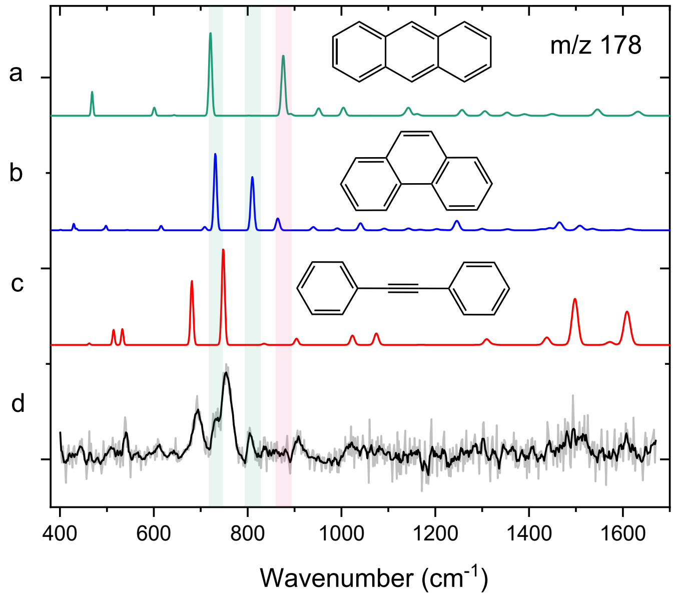

Supplementary Figure 10: Comparison of calculated infrared spectra of possible isomers for the m/z 178 channel to the experimental IR spectrum. The calculated spectrum of diphenylacetylene (red, c) is assigned to the experimental spectrum of m/z 178 (black, d). A small contribution of phenanthrene (blue, b) can be observed, highlighted by the light-green shaded bands. The presence of anthracene (green, a) can be excluded by the missing band shown in the light-red shaded band.

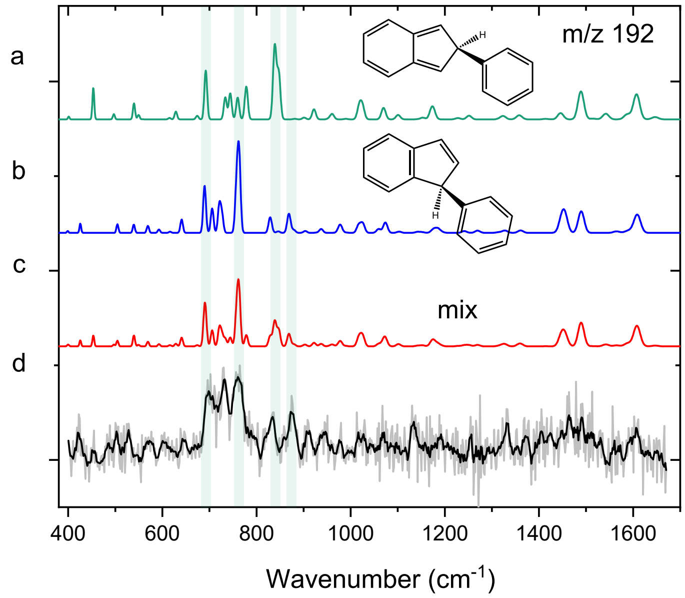

Supplementary Figure 11: Comparison of calculated infrared spectra of possible isomers for the m/z 192 channel to the experimental IR spectrum. The calculated spectra of 1-phenyl-1H-indene (blue, b) and 2-phenyl-2H-indene (green, a) show a good agreement with the experimental spectrum (light-green shaded bands). A mixture of 1-phenyl-1H-indene (2/3) and 2-phenyl-2H-indene (1/3) is shown in red and is assigned to the experimental IR spectrum of m/z 192. The presence of more isomers cannot be excluded and the assignment of solely of 1-phenyl-1H-indene (2/3) and 2-phenyl-2H-indene is therefore tentative.

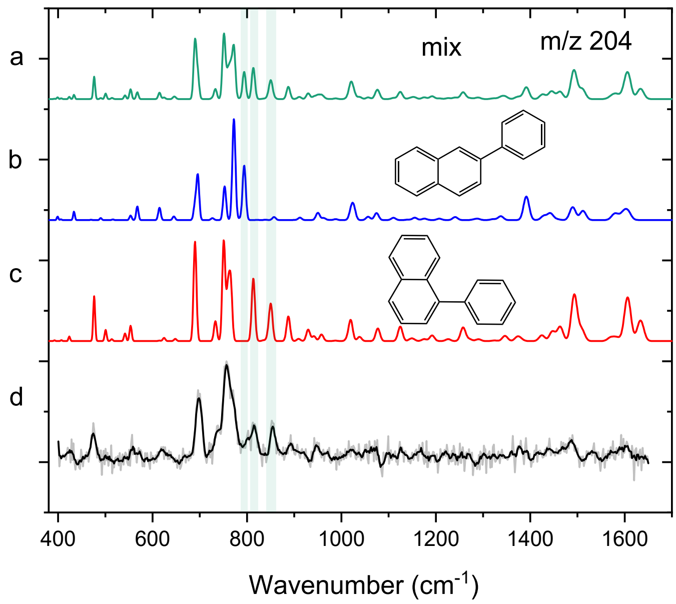

Supplementary Figure 12: Comparison of calculated infrared spectra of possible isomers for the m/z 204 channel to the experimental IR spectrum. The calculated spectrum of 1-phenylnaphthalene (red, c) is assigned to the experimental spectrum of m/z 204 (black, d). The presence of 2-phenylnaphthalene (blue, b) is proven by the experimental intensity highlighted in the light-green shaded band. A 1:1 mix of 1 and 2-phenylnaphthalene provides a more spectrally diluted intensity distribution in the high wavenumber region (>1000 cm^{-1}), and therefore a more accurate match with the experimental spectrum.

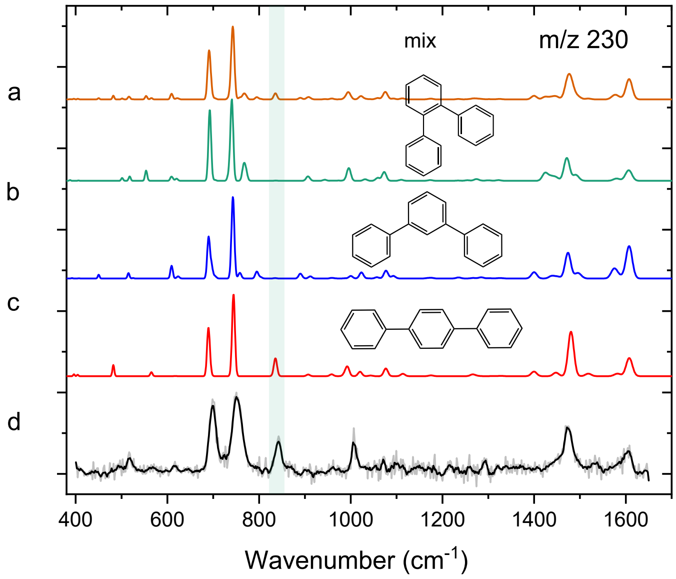

Supplementary Figure 13: Comparison of calculated infrared spectra of possible isomers for the m/z 230 channel to the experimental IR spectrum. The calculated spectrum of p-terphenyl (red, c) is assigned to the experimental spectrum of m/z 230 (black, d). Contributions of m-terphenyl and o-terphenyl are excluded based on the missing diagnostic band attributed to the p-terphenyl molecule (light-green shaded band). A combination of isomers would result in a decrease in intensity of this band, not in line with the experimental intensity.

Additional reaction pathway for the formation of tropyl-acetylene via the phenyl radical

Based on the many identified reaction products from the PAC class, the phenyl radical may also be a possible precursor to form the tropylacetylene (**B-4**) product. Multiple formation pathways from the phenyl radical with acetylene (**P**) are shown in Supplementary Figure 14. The barrier for H removal (**TS-P-1**) from the phenyl-acetylene adduct (**P-1**) is slightly above zero (+ 2.1 kJ/mol) and this will form phenylacetylene (**B1-2**). We do not observe the phenyl-acetylene adduct (**P-1**) intermediate and therefore suspect that this small barrier does not play a significant role in our experiments.

Additionally, we have also investigated the reactions of benzene with the methylidyne radical and subsequent addition with acetylene. This pathway is found to be overall endothermic by +35 kJ/mol with respect to the tropylacetylene product and is therefore suspected to play a minor role compared to the reaction starting with an ethynyl radical.

In all calculated sequences, the bicyclic three-membered ring structures and their low-energy transition states play a role in the ring expansion mechanisms. No spectroscopic signatures of the bicyclic three-membered ring intermediates were observed in the present experiment. Calculations on the potential energy surfaces of these ring growth mechanisms of the larger PAHs aromatics naphthalene and pyrene point towards energy barriers of 11 and 30 kJ/mol, respectively, thereby increasing the possibility of observing these intermediates in the molecular beam. The small methylidyne radical, detected in high abundances in different regions of the ISM[4], is found to be a key player in these ring expansion mechanisms of PAHs.

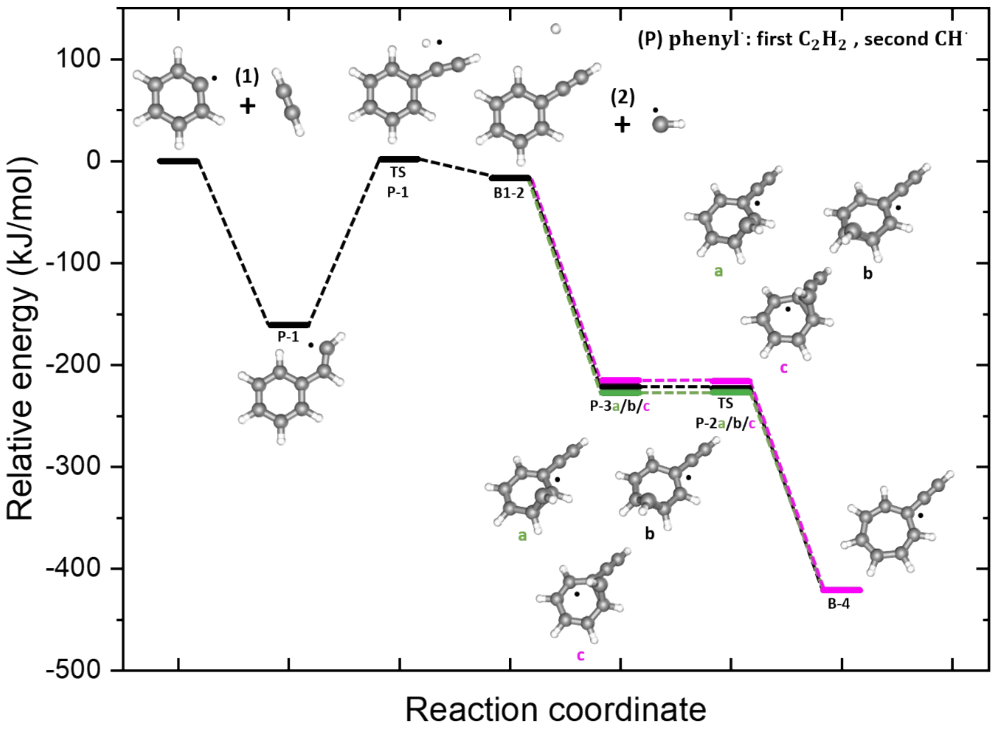

Supplementary Figure 14: Potential energy surfaces of the phenyl radical + acetylene + methylidyne radical reaction pathways to tropyl-acetylene radical. The main pathway from the phenyl radical with acetylene and methylidyne radical is shown in black. The three routes involving the different bicyclic intermediates are colored and denoted by a,b,c. The energies include the zero point vibrational energy correction and are calculated with respect to the entrance energy.

References

1. Constantinidis, P., Hirsch, F., Fischer, I., Dey, A. & Rijs, A. M. Products of the Propargyl Self-Reaction at High Temperatures Investigated by IR/UV Ion Dip Spectroscopy. *J. Phys. Chem. A* **121**, 181–191 (2017).

2. Nyquist, R. A. & Potts, W. J. Infrared absorptions characteristic of the terminal acetylenic group (-C≡C-H). *Spectrochim. Acta* **16**, 419–427 (1960).

3. Lemmens, A. K., Rap, D. B., Thunnissen, J. M. M., Willemsen, B. & Rijs, A. M. Polycyclic aromatic hydrocarbon formation chemistry in a plasma jet revealed by IR-UV action spectroscopy. *Nat. Commun.* **11**, 269 (2020).

4. Stutzki, J. & Wyrowski, F. Unveiling the chemistry of interstellar CH. *Astron. Astrophys.* **612**, A37 (2018).
